# Moderating Effect of the Situation of Return or Relocation on the Well-Being and Psychosocial Trauma of Young Victims of the Armed Conflict in Colombia

**DOI:** 10.3389/fpsyg.2020.603964

**Published:** 2020-12-21

**Authors:** Sandra Milena Quintero-González, Camilo Alberto Madariaga-Orozco, Anthony Constant Millán-de Lange, Diany Marcela Castellar-Jiménez, Jorge Enrique Palacio-Sañudo

**Affiliations:** Departamento de Psicología, Universidad del Norte, Barranquilla, Colombia

**Keywords:** well-being, psychosocial trauma, youth, victims, repair, moderating effect, armed conflict

## Abstract

Colombia is the second country with the highest number of internally displaced persons. In the last 10 years, more than 400,000 young people carry, in their life experiences, the title of victims. The psychological and social circumstances that determine the lives of displaced young people in the world are not unknown. Fear, the poor resources for social adaptation available to them, and the possible reproduction of the cycle of violence, represent psychosocial risk factors in the young and displaced population. In this context, the Victims Law in Colombia stipulated various measures of repairment, including Relocation (the person or household victim of forced displacement decides to settle in some place, other than the one they were forced to leave) and Return (the person or the household victim of forced displacement decides to return to the place from which they were displaced, in order to settle indefinitely) provided the conditions of voluntariness, security, and dignity are present. A hypothesis that well-being will be better in the returnees was set, since they would strengthen the social support networks between neighbors and other victims in their old spaces of life. To test the hypothesis, the scales of Psychological Well-being, Social Well-being, the Satisfaction with Life Scale, and the Psychosocial Trauma Scale were applied to young returnees (*n* = 129) and relocated (*n* = 259) in Colombia. The Exploratory and Confirmatory Factor Analysis was performed to extract the general measure of well-being and psychosocial trauma followed by the comparison between the groups. Significance, power, and effect size indicators were obtained, and finally, the partial correlation between the groups was made in relation to psychosocial trauma and well-being. Results showed that returnees have greater well-being and clearer indicators (*d* = 0.365, 1-β = 0.996), with respect to that of relocated. In addition, the well-being of returnees has fewer trauma factors, who in turn are quasi-moderated by the situation of return or relocation.

## Introduction

For 4 years in a row, Colombia has ranked second on the list of countries with the highest number of displaced people in the world (Alto Comisionado de las Naciones Unidas para los Refugiados [ACNUR], 2019). To date, there are more than 8 million forcibly displaced persons, including 3 million youth, of which 17,728 have returned and/or relocated between 2010 and 2018 (Unidad de Atención y Reparación Integral a las Víctimas [UARIV], 2020). History marks them: the more than 60 years of violence caused by a sociopolitical conflict that has not ceased. Collective violence is a phenomenon of great instrumental and emotional impact (Arendt, 2005; Blanco et al., 2016). The depth of the sequelae generated by this phenomenon has strong repercussions at an individual level, in terms of the destruction of a life project, and at a collective level, in the destruction or reformulation of social and support networks (Thompson and Walsh, 2010).

The Law in Colombia has established the restitution of rights to restore, to the greatest degree possible, the state of life in which people were prior to their forced displacement, (Ley de 387, 1997, chapter II; Ley de 1448, 2011, chapter II; Unidad de Atención y Reparación Integral a las Víctimas [UARIV], 2020). They have two options under the principles of security, voluntariness, and dignity: One is to return, if their decision is aimed at reintegrating to the place where they were forced to leave through the use of violence; and another is to relocate, if they choose to rebuild their life in a place other than the place of origin (Ley de 387, 1997, Chapter II). Which of the two conditions could be more favorable for the displaced, in light of its impact on well-being and the psychosocial trauma they have experienced?

The concept of return began to be studied at the end of the Second World War (Black and Gent, 2006). South Sudan, Bosnia and Herzegovina, Uganda, and Afghanistan appear as the countries with the highest returns, within and toward them, with loss of confidence and doubts about their own capacities, destruction of homes, despair, a pessimistic view of the world, among others, influencing the decision to return or to relocate (Bozzoli et al., 2012; Haroz et al., 2013; Huser et al., 2019; Sullivan, 2019; Renner et al., 2020), and affect their well-being (Siriwardhana and Stewart, 2013). Various international studies have focused on this population in order to better understand the consequences of return and relocation on the well-being of these victims (Diener and Diener, 1995; Black and Gent, 2006; Siriwardhana and Stewart, 2013; Burns et al., 2018).

Despite the fact that, in Colombia, research has been developed in relation to forced displacement, the scientific study of return and relocation is recent, and perhaps this leads to limited scientific and empirical evidence from the country (Ibáñez and Querubín, 2004; Hernández, 2010; Garzón, 2011; Siriwardhana and Stewart, 2013), especially the experience of trauma and well-being in the younger population. Some qualitative studies or bibliographic reviews highlight that situations of violence, previously unfounded terrors, and threats of being displaced again, become elements that hinder return processes (Lima and Reed, 2000; Chávez and Falla, 2005; Correa De Andreis, 2009; Garzón, 2011). Returning to the place of origin does not only imply a return, but a reconstruction and re-significance of the territory, a desire to re-have the experiences they lived there, linking affective components, including identity, in the territory where they would have already configured their life project (Posada et al., 2013; Velásquez and Céspedes, 2019).

The communities formed in new spaces are not natural or spontaneous, but created by administrative acts of the State, to comply with the law, rather than in the sense of comprehensive reparation to the victims (Arango and Arroyave, 2017). The traumatic experience can transform the structure of values and beliefs in different areas (personal, family, social) (Medina-Montañez et al., 2007; Blanco et al., 2016), and it differs according to groups and social classes (Madariaga, 2002).

The concept of psychosocial trauma has historically been approached from different perspectives, disciplines, and contexts (Freud, 1948; Chía-Chávez et al., 2011; American Psychiatric Association [APA], 2013; Bohigas et al., 2015). In the 20th century, the psychosocial period of trauma began, under the gaze of historical trauma (Bohigas et al., 2015). In the 90s, Ignacio Martín-Baró placed it in the Latin American context as “Psychosocial Trauma,” and defined it as “the crystallization–or materialization–in individuals of aberrant and dehumanizing social relationship such as those that prevail in situations of civil war” (Martín Baró et al., 1990, p. 236). Martín-Baró also identifies resources and positive elements in this experience of violence, which result from the need to continue life, since “the injury or affectation will depend on the peculiar experience of each individual, an experience conditioned by their social extraction, by their degree of participation in the conflict, as well as other characteristics of his personality and experience” (Martín Baró, 1990, p. 10). As main characteristics or factors that make up this concept of psychosocial trauma, the following are proposed: (1) the pre-traumatic situation referred to the causes of the generated trauma; (2) the personal, family, and community destruction as a consequence of the traumatic event; (3) disintegration of the inner world, the disintegration of beliefs about oneself, about others, and society; (4) intergroup emotions as emotional reactions referring to oneself, their community, and to the “others” (victimizers); (5) expression of emotional ambivalence such as the coexistence of positive and negative emotions toward the trauma; (6) recognition of personal capacities to overcome trauma; and finally (7) personal and collective efficacy (Blanco et al., 2014; Quintero, 2020 in press).

The experience of violence in Colombia carries the cross of trauma in a totally dehumanized and cold context. Enough research has studied the painful effect of collective violence on the displaced, demonstrating the destruction of territories of life, communal identities (Correa De Andreis, 2009; Cardozo Rusinque et al., 2017); post-traumatic stress disorders (PTSD), mood disorders (Aristizábal et al., 2012), detrimental quality of life, social media breakdown, modification of family roles, and family disintegration (Bell et al., 2012; Charry-Lozano, 2016; Sánchez et al., 2019).

Different authors affirm that the condition of returning or relocating can be, in itself, a traumatic event, especially after a prolonged displacement. The social and cultural ties established with the new place of settlement can be broken, feelings of helplessness and distrust appear, and the new generations that were born after displacement may have difficulties in adapting to an unknown place (Siriwardhana and Stewart, 2013; Arévalo, 2016; Sánchez et al., 2019). Either of these two ways requires the reconstruction of their well-being, so understanding trauma during this reintegration is complex (De Smet et al., 2019; Huser et al., 2019).

Positive Psychology at the end of the 90s has been in charge of precisely this: recognizing the positive and well-being aspects that are a fundamental part of human life, despite the painful experiences that life offers (Seligman and Csikzentmihalyi, 2001). As it is known, the first conceptualizations about well-being had their origin in the philosophical reflections of two traditions: the Eudaimonic Tradition, associated with individual and social human development (Ryff, 1989; Keyes, 1998); and the Hedonic Tradition, related to the effects and cognitions of an individual with respect to their own life (Diener, 1984; Keyes et al., 2002).

Indeed, well-being is closely linked to psychological and social experience (Seligman and Csikzentmihalyi, 2001; Keyes, 2005). The complete state of health model proposed by Keyes (2005), measured with the variables of psychological and social well-being and life satisfaction, has been widely used in research with victims of displacement in Colombia (Palacio et al., 1999; Palacio and Sabatier, 2002; Manrique et al., 2008; Abello–Llanos et al., 2009; Campo-Arias and Herazo, 2014; Amaris et al., 2019). However, a large number of researchers have concluded that it is possible to measure the well-being construct from a general score that underlies the scores of these variables as a whole (Keyes, 2005; Díaz et al., 2007; Kokko et al., 2013; Hides et al., 2016; Peña Contreras et al., 2017).

Along these lines, and given the existence of multiple instruments that measure well-being (Diener et al., 1985; Ryff, 1989; Ryff and Keyes, 1995; Keyes, 2005; Sisask et al., 2008; McDowell, 2010; Schneider et al., 2010; Kokko et al., 2013, p. 110; Hides et al., 2016; Peña Contreras et al., 2017) it became necessary to have a methodology that would allow a unified measure of well-being from different sources of record. Therefore, in this study, a factorial analysis that allowed finding a common structure between them was carried out. Based on these results, it was determined that the construct of “general well-being” describes the positive functioning of the well-being of a person in their psychological, social, and subjective domains (Keyes, 2005; Castellar, 2020, in press).

Not knowing and not addressing the experience of pain and suffering of young returnees and relocated, prevents the recognition of the impact and effectiveness of their condition of reparation (whether as returned or as relocated) for the reconstruction of their life project and well-being (Rebolledo and Rondón, 2010). This is why the relationship between psychosocial trauma and well-being is possibly moderated by the situation of return or relocation of the young victims. Starting from the difference between moderation and mediation in psychological studies (Baron and Kenny, 1986; Wen et al., 2005; Hayes, 2018) the experience of return or relocation can determine the effect of interaction in the relationship between psychosocial trauma and wellness. Identifying the moderating effect of this variable will allow researchers/professionals to plan their interventions to maximize the levels of well-being of the population (Internal Displacement Monitoring Centre [IDMC], 2020). To test its effect, a hypothesis is proposed: well-being and trauma are different according to the type of situation (return or relocation) of the young person.

## Materials and Methods

### Design

A retrospective ex-post-facto design was used, with the dependent variable (general well-being) being initially studied, and then independent variables (psychosocial trauma factors) being tested (Montero and León, 2007).

### Sample

The universe was made up of young victims of forced displacement, returned or relocated in the departments of Atlántico, Sucre, and Cesar (northern Colombia). The following was used as inclusion criteria: (1) that the participants were registered in the Single Registry of Victims (RUV). (2) That they were linked to return or relocation programs. (3) That they expressed willingness to participate. As an exclusion criterion, the category “Youth” in Colombia describes those persons whose ages range between 14 and 26 years of age, as established in Ley de 387 (1997). However, although they are young, all minors were excluded.

According to the RUV, in 2018 there were 8,808 young people returned or relocated in those three departments (Unidad de Atención y Reparación Integral a las Víctimas [UARIV], 2018). A sample of 369 subjects was selected, according to the representativeness criterion of 5% maximum admissible error and 95% confidence. Through non-probabilistic and incidental sampling, 388 participants of legal age were selected, given the self-selection bias that this entails in voluntary participation research (Kerlinger and Lee, 2002). According to gender, which is the only sociodemographic information registered in the RUV (36.67% men and 60.33% women), there were no statistically significant differences (with 95% confidence) between the characteristics of the population and the sample based on it (36.43% men, 63.57 women; *Z* = 1.27). The distribution of the subjects was as follows: 35% of the participants were in Atlántico, 25% in Cesar, and 41% in Sucre. 67% were in a relocation situation, and 33% in return. The ages were between 17 and 30 years of age (*M* = 23.5 and *D* = 4.183) of which, 78% were between 18 to 25 years, 19% were between 26 and 30 years.

### Technics and Instruments

#### Psychosocial Trauma Scale–ETAPS

A scale designed and validated with Colombian population by Villagrán (2016), composed of 62 items, which are originally grouped into four factors, namely: (1) the pre-traumatic situation, (2) destruction of sociality (3) personal and collective efficacy and (4) intergroup emotions. Following the recommendation of Villagrán (2016), in the context of the victim population in Colombia, Quintero (2020), (in press) determined the following structure with the same number of items presented as statements: (1) pre-traumatic situation, composed of 21 items (for example, “saying what I thought was about to cost me my life”), (2) personal, family, and community destruction, with nine items (for example, “living together with my family has become more difficult every day”) (3) disintegration of the inner world, with seven items (for example, “I have no one to count on”), (4) Intergroup emotions, made up of seven items (for example, “there can be no forgiveness for executioners”), (5) expression of emotional ambivalence, with six items (for example, “despite of what happened, I do not lose hope for the future”), (6) recognition of personal abilities, with four items (for example, “I have more confidence in myself”) and (7) personal and collective efficacy, composed of eight items (for example, “in my community/neighborhood, the participation of people in community activities has increased”). The response format ranges from 1 = “strongly disagree” to 7 = “strongly agree.” According to the analysis carried out on this sample, it can be pointed out that it is a factorially valid [χ^2^ = 6565.07, *p*-value = 0.000, root mean square error approximation (RMSEA) = 0.083, and adjusted goodness of fit index (AGFI) = 0.90], and reliable structure (Ω = 0.97). The scores were calculated by the refined regression method (DiStefano et al., 2009), for which the standardized scores (*M* = 0 and *D* = 1) are presented.

#### Ryff Scale of Psychological Well-Being

Scale designed by Ryff (1989), adapted and validated into Spanish by Díaz et al. (2006) composed of 29 items, with six dimensions originally distributed as follows: (1) Self-acceptance, (2) Positive relationships with other people, (3) Autonomy, (4) Environmental Mastery, (5) Purpose in life, and (6) Personal growth. For the context of the population victim of the armed conflict in Colombia, Quintero (2020), (in press) determined the following structure composed of the same number of items presented as affirmations: (1) Self-acceptance, with eight items (for example, For me, the life has been a continuous process of study, change, and growth), (2) ineffective relationships, with seven items (for example, I often feel lonely because I have few close friends with whom to share my concerns), (3) Personal planning, composed of seven items (for example, I enjoy making plans for the future and working to make them come true), (4) Relationships solid / strong interpersonal, with three items (for example, I feel that my friends bring me many things), (5) Self-assertion difficulty with two items (for example, I tend to worry about what other people think of me) and (6) Personal growth was composed of two items (for example, “I think that over the years I have not improved much as a person”). The answer options range from 1 = “strongly disagree” to 6 = “strongly agree.” According to the analyses carried out based on this sample, it can be pointed out: this structure is factorially valid (χ^2^ = 1209.94, *p*-value = 0.00000, RMSEA = 0.073, and AGFI = 0.94) and reliable (Ω = 0.96). The scores were calculated by the refined regression method (DiStefano et al., 2009), so the standardized scores (*M* = 0 and *D* = 1) are presented.

#### The Satisfaction With Life Scale

Proposed by Diener et al. (1985), SWLS, was adapted and validated into Spanish by Atienza et al. (2000), in a unidimensional way, consisting of five items. For the context of the population victim of the armed conflict in Colombia, Quintero (2020), (in press) determined the following structure composed of the same number of items presented as affirmations: (1) satisfaction with current life, with three items (for example, “the type of life I lead is similar to the type of life I always dreamed of leading”) and (2) satisfaction with the past life, with two items (for example, “so far I have obtained the important things I want in life”). The response format goes from 1 = “strongly agree” to 5 = “strongly disagree.” According to the analyses based on this sample, it can be pointed out that: this structure is factorially valid (χ^2^ = 3.55, *p*-value = 0.47, RMSEA = 0.000, and AGFI = 0.99) and reliable (Ω = 0.95). The scores were calculated by the refined regression method (DiStefano et al., 2009), therefore the standardized scores (*M* = 0 and *D* = 1) are presented.

#### Social Well-Being Scale

Constructed by Keyes (1998), and adapted to Spanish by Blanco and Díaz (2005) with 25 items in five dimensions distributed as follows: (1) social integration, (2) social acceptance, (3) social contribution, (4) social actualization, and (5) social coherence. For the context of the population victim of the armed conflict in Colombia, Quintero (2020), (in press) determined the following structure made up of the same number of items presented as statements: (1) social contribution, with eight items (for example, “I feel that I am an important part of my community”); (2) distrust of people, with three items (for example, “I think people are not to be trusted”); (3) anomie, with five items (for example, “many cultures are so strange that I cannot understand them”); and (4) distrust in the society development, with nine items (for example, “for me, social progress is something that does not exist”). The response format goes from 1 = “strongly agree” to 7 = “strongly disagree.” According to the analysis carried out based on this sample, it can be noted: this structure is factorially valid (χ^2^ = 719.43, *p*-value = 0.00000, RMSEA = 0.065, and AGFI = 0.90) and reliable (Ω = 0.94). The scores were calculated by the refined regression method (DiStefano et al., 2009), so the standardized scores (*M* = 0 and *D* = 1) are presented.

#### Sociodemographic Variables Booklet

It was prepared with the aim of finding out the sociodemographic characteristics of participants: age, sex, educational level, employment status, marital status, situation of comprehensive shelter (return or relocation).

### Procedure

After approval by the Ethics Committee of Universidad Del Norte, the participants were contacted through the professional team of the “*Unidad para la atención y reparación a las víctimas*” (Unit for Comprehensive Care and Reparation to Victims) of each department (Province), who voluntarily accepted and filled out the informed consent. The application of the booklet was carried out on paper, in the homes of the participants, without a time limit, and was assisted and guided by psychology professionals who were hired and trained on the variables of the study, the proper completion of the booklet, and the possible concerns or situations that could arise during the application. At its completion, a snack was given to compensate for their collaboration. Likewise, at the end of the collection of information, with the intention of corresponding ethically with the collaboration of the participants and, in recognition of the problems faced by the study population, workshops were held under the theme of achieving well-being. The application lasted approximately 6 months, after which the organization and information processing continued.

#### Data Analysis Plan

It began by confirming the existence of an underlying factorial structure, both in the model that considered the 12 well-being factors, if all the factors underlying each of the three tests used were considered; as in the model composed of only 8 of these factors, following the recommendations of Castellar (2020).

Subsequently, to determine which of the 2 one-dimensional structures should be used, the confirmatory factor analysis was applied, under the strategy of rival models (Hair et al., 2014), in order to determine, if all the scores of factorial factors of the 3 considered tests should be used, or if factors 2 and 3 of Social Well-being (Social Contribution and Distrust in the development of society), and factors 1 and 6 of Psychological Well-being (Self-acceptance and Difficulty for flexibility and obstinacy) should be dispensed of, following the recommendations of Castellar (2020).

Then, for internal consistency, the value of the Omega coefficient [Ω] was considered, which is the most suitable internal consistency coefficient for tests that were factorially validated (Heise and Bohrnstedt, 1970; McDonald, 1981; Muñiz, 1998; Ercan et al., 2007; Dunn et al., 2014) and higher than 0.70 points, according to the scale described by Prieto and Muñiz (2000) and updated by Hernández et al. (2016a, b). Next, the factorial score of all the participants was calculated, in the factor underlying the three well-being tests, to then analyze their compliance with the assumption of normality through the Kolmogorov-Smirnov Test [KS] for a sample, with correction of Lilliefors significance [K-SL].

To determine if there is any statistically significant difference between the common factor of general well-being and the factorial scores of psychosocial trauma, the Student’s *t*-test was used, whose interpretation was made according to the fulfillment of the homoscedasticity assumption, evaluated through the *p*-value of the Levene statistic. Likewise, to determine the size and magnitude of the effect (1-β), Cohen’s d-coefficient was used. Statistical power will be considered adequate, exceeding the 80% criterion.

The univariate correlation between the Psychosocial Trauma Factors was analyzed with respect to the common factor of general well-being, following the classification described by Prieto and Muñiz (2000), and Hernández et al. (2016a, b), to interpret the predictive relationship between psychological tests. Significance was evaluated with the 95% confidence criterion (α = 0.05). For the calculation of the relationship between the psychosocial trauma factors and the underlying general well-being, multiple regression was used by the method of steps forward, in order to determine which were the psychosocial trauma factors that were really related to well-being and maintaining compliance with the assumption of absence of multicollinearity, for this, those factors of psychosocial trauma whose tolerance measure was higher than the minimum expected criterion were excluded from the linear model of multiple regression (Minimum tolerance = 0.98). Likewise, compliance with the assumption of absence of multicollinearity was verified from the value of the coefficient of the Durbin-Watson [DW] indicator, which must be between the limits of 1.5 and 2.5 points. Subsequently, the direction of the effect of each of these psychosocial trauma factors incorporated into the multiple linear model were analyzed, based on the analysis of the signs of the regression coefficients [β].

In order to know the moderating effect of the returned and relocated variable, a multivariate statistical analysis model was used, which allowed for the estimation of the effect and the relationships between multiple variables. That is, considering that the quality of moderation implies “establishing differential levels of relationship” (Robles, 1997) between the variables, the value of the multiple correlation coefficient between the trauma factors identified in the multiple regression was compared, with respect to the common factor general well-being, but differentiated according to the situation of returnee or relocated; then, the methodology established by Baron and Kenny (1986), and, to determine the type of moderation, the guidelines established by Sharma et al. (1986) were followed and afterward, the magnitude of the differences in the change of the correlations with Cohen’s q-statistic, which will suppose a small difference, when its value is less than 0.20, a large difference, when it exceeds 0.5 points, and a median difference, when it is between both extremes. To estimate the *Z*-value of the differences between correlations, the Fisher Transform indicator was used; which allowed for the establishment of the existence of a statistically significant difference, when the absolute value of the difference exceeds the criterion of 1.96 points, or its *p*-value is less than 0.05 points.

Finally, the existence of the quality of moderation of the situation of returned or relocated was determined from the moderation analysis proposed by Hayes (2018), from the use of the Software Process (Hayes, 2018) that is incorporated into the SPSS. Statistically significant differences (Student’s *t*-test) were analyzed between the relocated victims compared to the returnees, in each of the trauma factors that maintain a statistically significant relationship with general well-being. In this way, the trauma factors that have relevant differences with respect to general well-being were determined, either independently of the type of situation (returnee or relocated), or in particular to one of the groups (returnees vs. relocated).

## Results

### Construction of the General Well-Being Score and Its Distribution

The multivariate normality assumption was fulfilled, both in model 1, of 12 well-being factors (RMK_1_ = 1,189), and in model 2, of eight factors (RMK_2_ = 1,167), suggested by Castellar (2020), (in press) and the existence of an underlying factorial structure will be determined in the case of model 2 (*d*_2_ = 0.504, KMO_2_ = 0.596 and Bartlett *p*-value_2_ < 0.001), but not so in Model 1 (*d*_1_ = 0.498, KMO_1_ = 0.385 and Bartlett *p*-value_1_ < 0.001).

**FIGURE 1:**
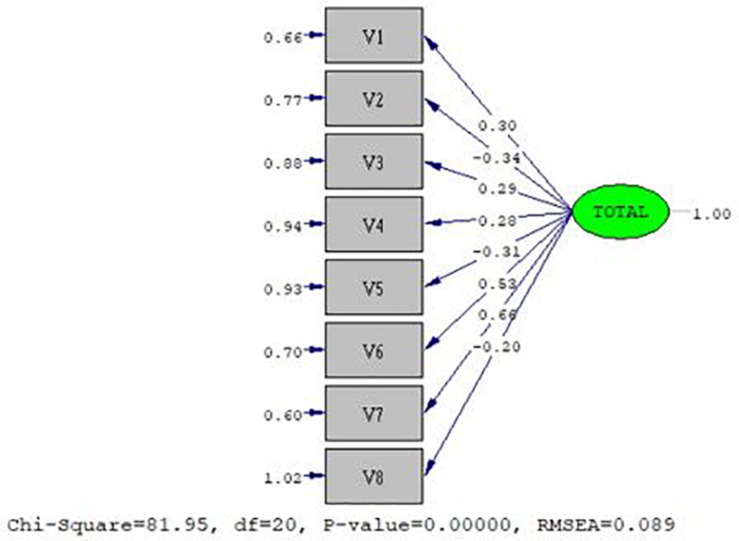
Representation of the best fit model.

According to the value of χ^2^, Model 2 could be considered as more appropriate than Model 1 (χ^2^_*Model* 2_ = 81.95 < χ^2^_*Model* 1_ = 155.08). However, it was not possible to distinguish which of the two models would best fit the data, based on the *p*-value of χ^2^, since in both cases, the value was *p* < 0,001. Although the goodness of fit index (GFI) of Model 2 (GFI_2_ = 0.95) was higher than that of Model 1 (GFI_1_ = 0.94), its difference was minimal. In the case of RMSEA, it could be determined that there was a better fit in the case of Model 2 (RMSEA_2_ = 0.089) compared to Model 1 (RMSEA_1_ = 0.070). As with the *p*-value of χ^2^, it was not possible to determine which model best fit the data based on the RMSEA *p*-value (RMSEA *p*-value_1_ = 0.00640 and RMSEA *p*-value_2_ = 0.0007). Based on the NCP and the ECV, it could be pointed out again that Model 2 has a better fit to the data (NCP_2_ = 61.950 and ECVI_2_ = 0.29), compared to Model 1 (NCP_1_ = 101.080 and ECVI_1_ = 0.52). It was not possible to determine the model with the best fit based on the AGFI, since the value was the same in both (AGFI_1_ = 0.91 and AGFI_2_ = 0.91). Finally, it was also concluded that Model 2 had a better fit to the data than Model 1, based on parsimony normed fit index (PNFI) (PNFI_1_ = 0.15; PNFI_2_ = 0.5), but not with the parsimony goodness of fit index (PGFI) (PGFI_1_ = 0.65; PGFI_2_ = 0.53). Model 2, in addition to being structurally valid, has adequate internal consistency (Ω = 0.776). All of the above allowed determining that Model 2 was the one that best fit the data collected in the three well-being instruments, and whose representation is presented in Figure 1.

Model 2 is made up of: from Factor 1 of Social Well-being, distrust in people [V1] and anomie [V2]. From Satisfaction with Life, there is satisfaction with current life [V3] and with past life [V4]; and Psychological Well-being, there is Factor 2 of ineffective relationships [V5], Factor 3 of planning life [V6], Factor 4 of solid/strong interpersonal relationships [V7], and Factor 5 on self-affirmation difficulty [V8].

**FIGURE 2:**
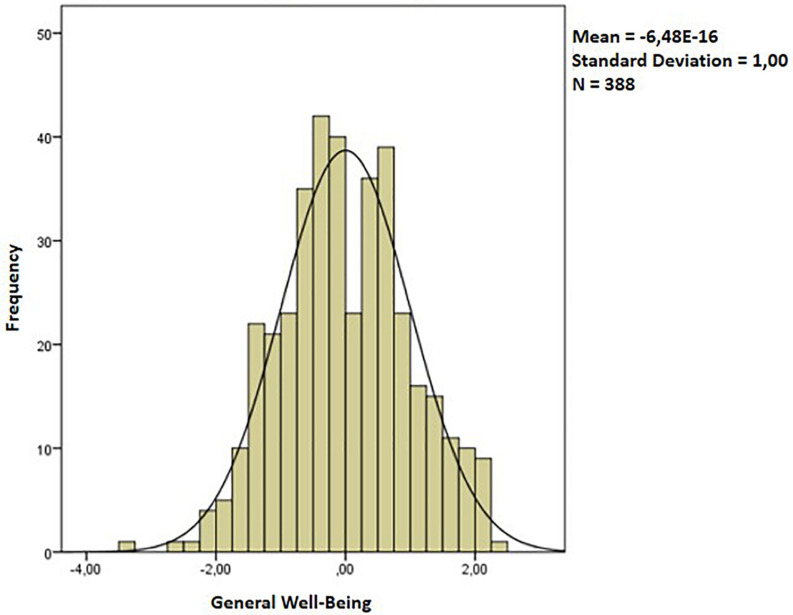
Distribution of the general well-being measure.

The shape of the distribution of this underlying measure of common general well-being (see Figure 2), assumes that its range covers the scores of −3.42 as a minimum value and a maximum of 2.25, with an average of *p* < 0,001 and a deviation of 1.00, which assumes that it is a standardized distribution that is also normal (K-S_*L*_ = 0.05).

### Comparing Returnees and Relocated With General Well-Being

Differences (*t* = −4.35) are statistically significant (*p*-value < 0.001), moderate (*d*_*Cohen*_ = 0.465) and strong (1-β = 0.996) in favor of returnees (*X*_*R*__*eturnees*_ = 0.31; *S*_*returnees*_ = 1.01), in comparison with the relocated (*X*_*R*__*elocated*_ = −0.15; *S*_*relocated*_ = 0.96), after having estimated these differences, considering compliance with the homoscedasticity assumption (F-Levene = 0.63; *p*-value = 0.43).

### Comparing Returned and Relocated With Psychosocial Trauma Factors

In Table 1, statistically significant differences are observed between the situation of returnees vs. relocated between: (1) pre-traumatic situation (*p*-value < 0.001, *d*_*Cohen*_ = 0.39, 1-β = 0.98), (2) disintegration of the inner world (*p*-value < 0.001, *d*_*Cohen*_ = 0.41, 1-β = 0.98), (3) intergroup emotions (*p*-value < 0.001, *d*_*Cohen*_ = 0.73, 1-β = 1.00), (4) expression of emotional ambivalence (*p*-value < 0.001, *d*_*Cohen*_ = 0.35, 1-β = 0.95), and (5) personal and collective efficacy (*p*-value < 0.001, *d*_*Cohen*_ = 0.44, 1-β = 0.99); which would suppose that to the extent that a participant belonged to the group of returned victims, a higher score would be expected for: pre-traumatic Situation (*X*_*R*__*eturnees*_ = 0.23; *S*_*returnees*_ = 0.94), intergroup emotions (*X*_*R*__*eturnees*_ = 0.45; *S*_*returnees*_ = 0.91), and personal and collective efficacy (*X*_*R*__*eturnees*_ = 0.31; *S*_*Returnees*_ = 0.93), while it is expected to have a lower score of: disintegration of the inner world (*X*_*R*__*eturnees*_ = −0.25; *S*_*returnees*_ = 0.78) and expression of emotional ambivalence (*X*_*R*__*eturnees*_ = −0.22; *S*_*returnees*_ = 0.99); on the other hand, if it belonged to the group of relocated victims, an opposite pattern would be expected in all these variables. Finally, no differences are observed between the measure of comprehensive reparation of the participants and the factors of personal, family, and community destruction (*p*-value = 0.18; *d*_*Cohen*_ = 0.14; 1-β = 0.37), and recognition of personal capacities (*p*-value = 0.95; *d*_*Cohen*_ = 0.01; 1-β = 0.06).

**TABLE 1:** Comparing returned and relocated with psychosocial trauma factors.

Group statistics							Levene test		*t*-test				
Trauma* Situation* Model*			*N*	Average	SD	Mean standard error	*F*	Sig.	*t*	df	Sig. (bilateral)	d effect size	Power (1-β)
Pre-traumatic situation	N*	ReL	259	−0.15	1.01	0.06	036	0.55	−3.62	386.00	0.00	0.39	0.98
		ReT	129	0.23	0.94	0.08							
Personal, family, and community destruction	Y*	ReL	259	−0.05	1.02	0.06	7.01	0.01	−1.36	300.91	0.18	0.14	0.37
		ReT	129	0.08	0.85	0.08							
Desintegration of inner world	Y	ReL	259	0.13	1.08	0.07	6.81	0.01	3.99	338.37	0.00	0.41	0.98
		ReT	129	−0.25	0.78	0.07							
Intergroup emotions	N	ReL	259	−0.23	0.94	0.06	0.00	0.98	−6.78	386.00	0.00	0.73	1.00
		ReT	129	0.45	0.91	0.08							
Expression of emotional ambivalence	Y	ReL	259	0.12	0.93	0.06	0.62	0.43	3.30	386.00	0.00	0.35	0.95
		ReT	129	−0.22	0.99	0.09							
Recognition of personal capacities	Y	ReL	259	0.03	1.06	0.07	7.50	0.01	−0.06	320.01	0.95	0.01	0.06
		ReT	129	0.04	0.82	0.07							
Personal and collective efficacy	Y	ReL	259	−0.12	1.00	0.06	4.00	0.05	−4.13	271.08	0.00	0.44	0.99
		ReT	129	0.31	0.93	0.08							

*Not a part of regression model (N); Part of regression model (Y). Relocated (ReL); Returnees (ReT).

### Correlation Between General Well-Being and Psychosocial Trauma Factors

It can be seen in Table 2, that the correlation between well-being and trauma factors are significant with an adequate score in 3 of them: personal, family and community destruction (*r* = −0.354; *p*-value < 0.001), disintegration of the inner world (*r* = −0.393; *p*-value < 0.001) with a negative score, and personal and collective efficacy (*r* = 0.357; *p*-value = 0.000) with a positive score. A sufficient relationship was also observed, but a little weaker, in the recognition of personal capacities (*r* = 0.203; *p*-value < 0.001). It is also observed that the direction observed in these relationships is coherent, since the higher the score in the personal, family, and community destruction and in the disintegration of the inner world, the lower the common general well-being score and the higher the score in the recognition of personal capacities and personal and collective efficacy, the greater common general well-being there will be. Likewise, it is observed that pre-traumatic situation (*r* = 0.008; *p*-value = 0.882) and intergroup emotions (*r* = −0.086; *p*-value = 0.092), maintain weak relationships, which are not statistically significant with general well-being, while the expression of emotional ambivalence (*r* = −0.119; *p*-value = 0.019), maintains a weak relationship, although statistically significant, with this same measure.

**TABLE 2:** Correlation between general well-being and psychosocial trauma factors.

	Pre-traumatic situation	Personal, family, and community destruction	Desintegration of inner world	Intergroup emotions	Expression of emotional ambivalence	Recognition of personal capacities	Personal and collective efficacy
Pearson correlation	0.008	−0.354	−0.393	−0.086	−0.119	0.203	0.357
Sig. (bilateral)	0.882	0.000	0.000	0.092	0.019	0.000	0.000

### Multiple Regression Between General Well-Being and Psychosocial Trauma Factors

The multiple relationship between the component factors of trauma and the general measure of well-being suppose an excellent and statistically significant relationship (*R* = 0.689; *p*-value < 0.001). This relationship supposes the fulfillment of the assumption of independence between the predictor variables (DW = 1.866). The following factors were excluded from this multiple linear model: (1) pre-traumatic situation and (2) intergroup emotions.

As seen in Table 3, the interpretation of the direction of the effects of each trauma factor is maintained, with respect to its prediction on the common factor of general well-being that was observed in Table 2 (bivariate); in this sense, the factors of personal, family, and community destruction; disintegration of the inner world; and expression of emotional ambivalence maintain an inverse relationship with the common factor of general well-being, while the factors recognition of personal abilities, and personal and collective efficacy maintain a direct relationship. In this table, it is also observed that the tolerance of these factors exceeds the minimum criterion of 0.98 points.

**TABLE 3:** Multiple regression between general well-being and psychosocial trauma factors.

	Non-standardized coefficients				Collinearity statistics			
	Beta	Standard error	*t*	Sig.	Tolerance	VIF	Δ Returnee vs. Relocated	Group with higher well-being**
(Constant)	−0.01	0.037	−0.371	0.711				
Desintegration of inner world	−0.41	0.037	−11.109	0.000	0.995	1.005	−0.25*	Returnees
Expression of emotional ambivalence	−0.13	0.038	−3.294	0.001	0.999	1.001	−0.22*	Returnees
Personal and collective efficacy	0.34	0.037	9.062	0.000	0.995	1.005	0.31*	Relocated
Personal, family, and community destruction	−0.38	0.038	−9.954	0.000	0.996	1.004	0.08	Both
Recognition of personal capacities	0.23	0.038	6.130	0.000	0.989	1.011	0.04	Both

*Significative difference. **Check the mean between the situation between returnees and relocated with general well-being.

### Quasi-Moderating Effect of the Situation of Return or Relocation With Psychosocial Trauma and General Well-Being

With regard to the quality of moderation of the return or relocation situation, an excellent statistically significant correlation was observed, both in the group of relocated participants (*R* = 0.615; *p*-value < 0.001) and in the returnees (*R* = 0.782; *p*-value < 0.001).

According to the moderation analysis (Hayes, 2018), it was possible to determine that there is no pure moderation effect between the psychosocial trauma factors (β = 0.66; *t* = 16.26; *p*-value = 0.000), and the returnee or relocated situation (β = −8.58; *t* = −2.11; *p*-value = 0.035) with respect to general well-being, since their interaction was not significant (β = 0.146; *t* = 1.793; *p*-value = 0.074). However, when analyzing the difference between both correlation coefficients, it could be observed that it was intermediate (*q*_*Cohen*_ = 0.334), statistically powerful (1-β = 0.922), and significant (*Z* = 3.014; *p*-value = 0.002), so it could be determined that the situation of returnee or relocated is a quasi-moderating variable (Sharma et al., 1986), since: (1) the situation of returnee or relocated of the participants, with respect to trauma factors was sufficient (*R* = 0.202) and statistically significant (*p*-value = 0.000) and (2) the situation of return or relocation of the participants, has a sufficient (*R* = 0.216) and statistically significant (*p*-value = 0.000) relationship with general well-being. Therefore, the situation of returnee or relocated could be classified as a quasi-moderating variable (Sharma et al., 1986) of the relationship between the statistical combination of psychosocial trauma factors and the common factor of general well-being.

The above, together with the information in Table 3 (If β_*Tabl*__*a*__3_ > 0→Reinforce; If β_*Tabl*__*a*__3_ < 0→Extinguish) and *X*_*Relocated*_ = −0.15; *S*_*relocated*_ = 0.96; *t* = −4.35; *p*-value = 0.000; *d*_*Cohen*_ = 0.46, allows for the determination of the trauma factors that should be intervened to improve the scores of the common factor of the general well-being of the victims of the Colombian armed conflict, regardless of whether the participant is relocated or returned (*p*-value > 0.05 and d_*Cohen*_ < 0.20), or, specifically about the relocated group, who have the lowest average score.

According to the above, it is necessary to reduce the effects of personal, family, and community destruction (β_*Tabl*__*a*__3_ = −0.38; *p*-value = 0.18; and *d*_*Cohen*_ = 0.14) while it is necessary to reinforce the effects of recognition of personal capabilities (β_*Tabl*__*a*__3_ = 0.23; *p*-value = 0.95; and *d*_*Cohen*_ = 0.01), in all participants, to improve their general well-being. On the other hand, for the relocated, it is necessary to strengthen personal and collective efficacy (β_*Tabl*__*a*__3_ = 0.34; *X*_*R*__*elocated*_ = 0.31; *S*_*relocated*_ = 0.93; *t* = −4.13; *p*-value = 0.00; and *d*_*Cohen*_ = 0.44), and reduce the disintegration of the inner world (β_*Tabla3*_ = −0.41; *X*_*R*__*elocated*_ = −0.25; *S*_*relocated*_ = 0.78; *t* = 3.99; *p*-value = 0.00; and *d*_*Cohen*_ = 0.41) and expression of emotional ambivalence (β_*Tabla3*_ = −0.13; *X*_*R*__*elocated*_ = −0.22; *S*_*relocated*_ = 0.99; *t* = 3.30; *p*-value = 0.00; and *d*_*Cohen*_ = 0.35), to improve their general well-being.

## Discussion

Well-being and psychosocial trauma in returnees and relocated are significantly different, so the hypothesis was not tested because there was no moderation, but rather quasi-moderation. General well-being (Summerfield, 2001; Lazarus, 2003; Keyes, 2005; Díaz et al., 2007; Castellar, 2020, in press) is higher in returnees, which is consistent with previous studies (Bozzoli et al., 2012; Ramírez, 2015; Arévalo, 2016; Mejía, 2019) who affirm that the construction of memory, social, and cultural identity at the scene of the events is essential for their well-being. This same process can be more difficult for the relocated, which makes them always feel displaced because they are in a place that is not their home (Palacio et al., 2003), maintaining a nostalgia for the past and a longing for what they were in their land. What about their life projects? How do they integrate into their new lives? Integration with their community is likely to overshadow the possible difficulties for returnees.

Following this argument, although a moderating effect was not observed, a quasi-moderating effect was observed due to the situation of returning or relocating, in the relationship between the factors of psychosocial trauma and well-being, which is corroborated by the statistically significant differences and mean size found when comparing the correlation coefficients of both groups. This means that the psychosocial trauma factors (disintegration of the inner world, personal, family, and community destruction, personal and collective efficacy, recognition of personal capacities, and expression of emotional ambivalence), however, they had a different relationship strength depending on the group they belong to: returnees or relocated.

In general terms, it was observed that to the extent that a person has a lower score in: disintegration of the inner world; personal, family, and community destruction; and expression of emotional ambivalence at the same time that they have higher scores of personal and collective efficacy, and recognition of personal abilities will have a higher overall wellness score. Notwithstanding the above, it will be stronger in the group of returnees in the following variables: disintegration of the inner world, and expression of emotional ambivalence. Although in the group of relocated it will be in the variable of: personal and collective efficiency. With this we contribute two new visions to the phenomenon of return and relocation in young people: (1) The facts and personal, family, and community experiences of both groups of young people have not been destroyed as previously thought, and (2) It is possible to recognize the personal capacities of young people in the environment they live in (be it returned or relocated). This is likely because young people have not been direct witnesses to the violence, leading them to perceive the situation of return or relocation as an opportunity to strengthen their personal, family, and community life. This result agrees with the exclusion of the pre-traumatic situation factor from the regression model for both groups.

Regarding the relationship between disintegration of the inner world and expression of emotional ambivalence in the general well-being of returnees, Ibáñez and Querubín (2004) showed that young people could consider a better experience of well-being in relocation, since they could have more opportunities of personal and social development. In contrast, the present study reveals that an adequate structure of attitudes and emotions associated with the experience of return shows in young people a significant indicator to maintain a high well-being, so that less destruction of the inner world will affect a greater well-being, contrary to what may happen to the relocated. Regarding the expression of emotional ambivalence, it is already confirmed by the General Report of the National Center for Historical Memory (Centro Nacional de Memoria Histórica, 2013) and in the study by Brewin et al. (1996), in which violent events are clear examples of traumatic experiences that they tend to destroy the belief, control, and meaning system, however, we propose that although they have marked their life, returning to their place of origin facilitates the recovery of their cognitive belief system, since it allows them to return to the way of life prior to displacement and with it increase their well-being.

Contrary to what is proposed by Hernández (2010), who compiles unemployment, precarious living conditions, and stigmatization for being victims in the stories of the relocated, this study reveals that despite the painful experience of displacement and the countless changes brought about by relocation, relocating has been able to change their personal and social life, recognizing that they are useful and effective. This is shown in their active participation in community activities, feeling a part of it.

In conclusion, psychosocial trauma cannot be defined outside of the historical and social context that surrounds it (Martín Baró et al., 1990), return and relocation have a particular power to shape the life history of human beings who choose it, perhaps as the institutional way to repair, but also for the motivation pursued every day to bring in a better well-being. Given that the study participants are young, the development of personal and collective capacities as a function of the constant search for their own well-being and that of their family is surprising, in the case of returnees, for example. This is a unique finding in this study, because the scientific generality addressed so far has shown the opposite (Haroz et al., 2013; Huser et al., 2019; Sullivan, 2019). In the relocated youth, despite the fact that they build a personal and social life, this is not enough to achieve high well-being, it seems that the effort to be made is greater than in the returnees.

As limitations of the study, it is necessary to mention that for this type of situation, it is difficult to carry out experimental studies given the difficulty in the ethical manipulation of the variables, and because of access to the participants. We realize that there are also limitations in the information available in the databases of the organizations that work with this population. Furthermore, it is necessary, for future studies, to take into account the following variables: (1) Travel time, return and relocation time. (2) Population indicators such as educational level, type of reparation (if relocated or returned). (3) Intensity of the victimizing acts (being threatened, beaten or injured, being a direct witness to murders, etc.). (4) Dearth of scientific studies on the effects of return and relocation in Colombia.

From the point of view of ensuring better precision in the prediction of well-being, it is recommended to continue with the study of other variables that make it possible to better predict the well-being of the relocated, such as the time spent at the relocation site or their sense of community. Social identity and memory would contribute greatly to know the process of rebuilding its social fabric. On the other hand, resilience and post-traumatic growth would be convenient to verify positive experiences that may be associated with overcoming trauma. In addition to psychosocial trauma, the variable of transgenerational transmission of trauma must be considered, since the intensity of memories of the experience of violence in the population participating in this study may influence this variable. It is also recommended to consider the variable length of stay for returnees, since it may be relevant to their well-being. This undoubtedly motivates the authors to carry out future research.

With a view toward a psychosocial intervention, a guide is proposed to the people in charge of its application to make decisions about the advantage of being able to propose strategies for return or permanence according to the general profile of the victims. The intervention would be more costly in the relocated than in the returnees, not only because their well-being is lower, but the variables that are related to it are less precise in this group. A variable that could be reinforced in young returnees with low levels of well-being would be personal and collective efficacy, through actions that lead to social skills training, for example. On the contrary, the intervention in the relocated youth would focus on minimizing the disintegration of the inner world and the expression of emotional ambivalence in order to maintain their personal and collective efficacy. For both groups, it is necessary to join forces in proposing activities that allow maintaining the experiences associated with the recognition of their personal capacities and reducing those associated with personal, family, and community destruction. Even though there was a statistically significant difference between returnees and relocated in the pre-traumatic situation factors and intergroup emotions, having not entered the regression models, no intervention planning based on these factors is justified, given that even when there are differences between groups, it does not generate any contribution to general well-being. The updating of public policies arises as a reflection on these results. An effort to redirect the focus of return and relocation programs as comprehensive reparation measures could have a better sustainable effect on the well-being of the participants.

## Data Availability Statement

The original contributions presented in the study are included in the article/supplementary material, further inquiries can be directed to the corresponding author/s.

## Ethics Statement

The studies involving human participants were reviewed and approved by Comité de Ética en investigación de la División Ciencias de la Salud de la Universidad del Norte. The patients/participants provided their written informed consent to participate in this study.

## Author Contributions

AM, SQ-G, CM-O, and JP-S contributed to the conception and design of the study. SQ-G organized the database. AM performed the statistical analysis. SQ-G and DC-J wrote the first draft of the manuscript. All authors contributed to the article and approved of the presented version.

## Conflict of Interest

The authors declare that the research was conducted in the absence of any commercial or financial relationships that could be construed as a potential conflict of interest.

## Abbreviations

ACNUR: United Nations High Commissioner for Refugees
UARIV: Unit for Comprehensive Attention and Reparation for Victims
KMO: Kaiser-Meyer-Olkin Measure
GFI: goodness of fit index
RMSEA: root mean square error approximation
ECVI: expected cross validation index
AGFI: adjusted goodness of fit index
PNFI: parsimony normed fit index
PGFI: parsimony goodness of fit index
K-S_*L*_: Lilliefors
DW: Durbin-Watson coefficient index
K-S: Kolmogorov-Smirnov test.
